# Engineering Ca-BTC MOF Nanorods with Electrochemical and Luminescent Functionalities

**DOI:** 10.3390/molecules31214035

**Published:** 2026-11-09

**Authors:** Luis A. Garcés-Patiño, Esmeralda L. Martínez-Piñeiro, Edith Osorio-de-la-Rosa, Nicolás Cayetano-Castro, Ulises Caldiño, Fernando Pérez-Villaseñor, Mirna Valdez-Hernández, Marisol Hernández-Gutiérrez, Cristian A. Ospina-DeLaCruz, Ángel Castro-Agüero, Arturo Ortiz-Arroyo, Abraham Pacio-Castillo, Dulce Y. Medina-Velázquez

**Affiliations:** 1 Advanced Materials Division, Potosino Institute for Scientific and Technological Research (IPICYT), San Luis Potosí 78216, Mexico; luis.garces@ipicyt.edu.mx; 2 Faculty of Sciences, National Autonomous University of Mexico (UNAM), Coyoacán, Mexico City 04510, Mexico; esmeraldamartinez@ciencias.unam.mx; 3 SECIHTI, Division of Sciences, Engineering, and Technology, Autonomous University of the State of Quintana Roo (UQROO), Chetumal 77019, Mexico; eosorio@secihti.mx; 4 Center for Nanosciences and Micro and Nanotechnologies (CNMN), National Polytechnic Institute (IPN), Gustavo A. Madero, Mexico City 07738, Mexico; ncayetanoc@ipn.mx; 5 Department of Physics, Metropolitan Autonomous University (UAM), Iztapalapa, Mexico City 09310, Mexico; cald@xanum.uam.mx; 6 Faculty of Basic Sciences, Engineering and Technology, Autonomous University of Tlaxcala (UATx), Apizaco 90401, Mexico; fernando.perez@uatx.mx (F.P.-V.); angel.castro@uatx.mx (Á.C.-A.); arturo.ortiz@uatx.mx (A.O.-A.); 7 Biodiversity Conservation Department, San Cristóbal de las Casas Campus, El Colegio de la Frontera Sur (ECOSUR), San Cristóbal de las Casas 29290, Mexico; mavaldez@ecosur.mx; 8 Center for Research in Semiconductor Devices, Institute of Sciences, Meritorious Autonomous University of Puebla (BUAP), Puebla 72570, Mexico; marisol.hernandez.g@uvp.edu.mx (M.H.-G.); pacio1904@gmail.com (A.P.-C.); 9 Computer Engineering Program, Polytechnic University of the State of Morelos (UPEMOR), Jiutepec 62574, Mexico; crisalod@gmail.com

**Keywords:** multifunctional materials, metal–organic frameworks, calcium-based MOF, luminescence, supercapacitors

## Abstract

Metal–organic frameworks (MOFs) have emerged as versatile materials for advanced applications due to their exceptional structural diversity, high surface areas, and tunable porosity. Among them, calcium-based MOFs (Ca-MOFs) are attracting significant interest owing to the natural abundance, low cost, and biocompatibility of calcium. In this work, a calcium benzene-1,3,5-tricarboxylate (Ca-BTC) MOF was successfully synthesized and characterized as a multifunctional material. X-ray diffraction confirmed the formation of the crystalline Ca_3_(BTC)_2_ phase, in agreement with the simulated pattern derived from the reported single-crystal structure. Electron microscopy revealed a nanorod morphology and ordered crystalline regions at the nanoscale, while Raman spectroscopy provided evidence of Ca–O coordination and the characteristic carboxylate bonding of the BTC linker. Under 365 nm excitation, the Ca-BTC MOF exhibited a bluish-white emission with significantly higher intensity than that obtained under 265 nm excitation, together with an average luminescence lifetime of 51.48 ms and a photoluminescence quantum yield of 7.7%. The higher emission intensity under near-UV excitation highlights its potential as a phosphor for pc-WLED applications. The electrochemical performance of Ca-BTC was evaluated in a solid-state supercapacitor (SC) assembled with graphene-based electrodes and a phosphoric acid electrolyte. Electrochemical measurements demonstrated a specific capacitance of 296.5 F g^−1^ at 25 mV s^−1^, together with excellent cycling stability, retaining 97.8% of its initial capacitance after 1000 charge–discharge cycles. These findings demonstrate that the Ca-BTC MOF combines a crystalline structure, long-lived bluish-white luminescence, and electrochemical charge-storage properties, making it a promising candidate for optoelectronic and energy-storage applications.

## 1. Introduction

The increasing global demand for advanced technologies is associated with two critical challenges: the need for efficient and sustainable energy storage and the dependence on rare-earth elements in optoelectronics. The depletion of fossil fuels and growing environmental concerns have increased the need for energy storage systems that offer high power density and long cycle life, making devices with high specific capacitance attractive candidates [1,2,3,4]. At the same time, the current dependence on rare-earth elements for luminescent materials used in solid-state lighting and displays presents important challenges. This dependence is associated with supply chain concerns, price fluctuations, and environmental impacts related to rare-earth extraction and processing. These factors, together with concerns about their ecological and toxicity effects, highlight the need for alternative materials and more sustainable technological approaches [5,6,7,8].

Metal–organic frameworks (MOFs) have emerged as versatile materials with the potential to address these challenges. Their high surface areas, tunable porosity, and adjustable chemical environments make them promising for energy-storage applications, particularly for improving ion transport and charge-storage capacity [2,9,10]. In addition, the use of different organic ligands provides opportunities to introduce luminescent and other functional properties. The combination of these properties gives MOFs potential for multifunctional applications, which can be influenced by their controlled structure and morphology, including nanorod morphologies [11,12,13,14]. In the search for more sustainable materials, calcium (Ca) is an attractive element for the development of MOFs. As the fifth most abundant element in the Earth’s crust, calcium is relatively low-cost and exhibits low toxicity and good biocompatibility [15,16]. Calcium-based MOFs (Ca-MOFs) have also been reported to show good stability in the presence of moisture, although their stability depends on the metal-ligand combination and framework structure [17,18].

Early work by Platers et al. [19] established an important structural basis for Ca–BTC materials through the hydrothermal synthesis and single-crystal X-ray characterization of Ca(Hbtc)·2H_2_O, revealing a layered structure in which Ca^2+^ ions are coordinated by carboxylate groups and water molecules. Notably, their study focused primarily on the synthesis and structural characterization of this compound and on its behavior during dehydration and rehydration, providing insight into the structural behavior of the calcium–carboxylate network [19]. Building upon this structural foundation, subsequent studies have demonstrated that appropriate ligand design can provide Ca-based coordination materials with interesting photophysical properties, offering potential alternatives to rare-earth-based luminescent systems [20,21,22,23]. However, the multifunctional integration of luminescence and electrochemical charge storage in Ca-BTC-based materials has received limited attention.

The present work builds upon these previous structural and synthetic studies by developing Ca-BTC MOF nanorods and evaluating their multifunctional properties. In contrast to previous studies focused primarily on the structural characterization or individual properties of Ca-based coordination materials, the present study investigates the combination of rare-earth-free bluish-white luminescence and electrochemical charge-storage functionality within the same Ca-BTC platform. The luminescent response demonstrates potential for applications in phosphor-converted white light-emitting diodes (pc-WLEDs), while the electrochemical results demonstrate notable capacitive performance [24,25]. Thus, the novelty of this work lies in the integration and evaluation of these two functionalities in Ca-BTC MOF nanorods, extending previous studies of Ca–BTC materials toward multifunctional luminescent and energy-storage applications.

## 2. Results and Discussion

### 2.1. Structural Characterization

**Figure 1:**
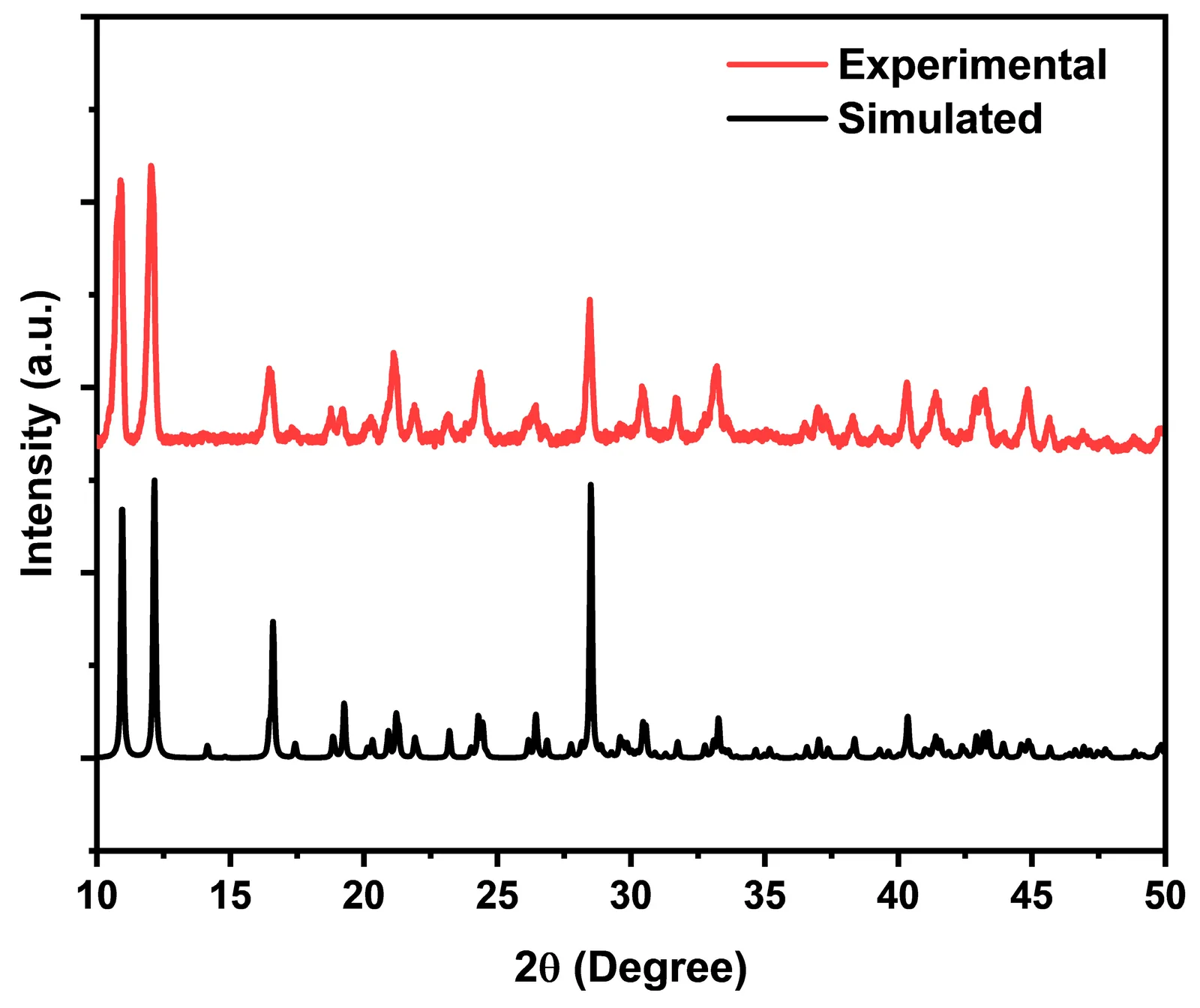
XRD pattern of the Ca-BTC MOF (red) and simulated XRD pattern of the Ca_3_(BTC)_2_ single-crystal structure (CCDC 1252228) (black).

Figure 1 presents the XRD pattern of the synthesized material and the simulated pattern based on the reported Ca_3_(BTC)_2_ single-crystal structure (CCDC 1252228) [19]. The simulation was performed using Mercury software (version 2026.1, Cambridge Crystallographic Data Centre, CCDC). The experimental diffraction peaks are in good agreement with those of the simulated pattern, supporting the formation of the Ca-BTC crystalline phase and indicating that the synthesized material is structurally consistent with the previously reported Ca-BTC framework. The data indicate that the framework exhibits slight lattice distortion, while the broadening of the diffraction peaks is consistent with the presence of small crystalline domains and local structural disorder.

**Figure 2:**
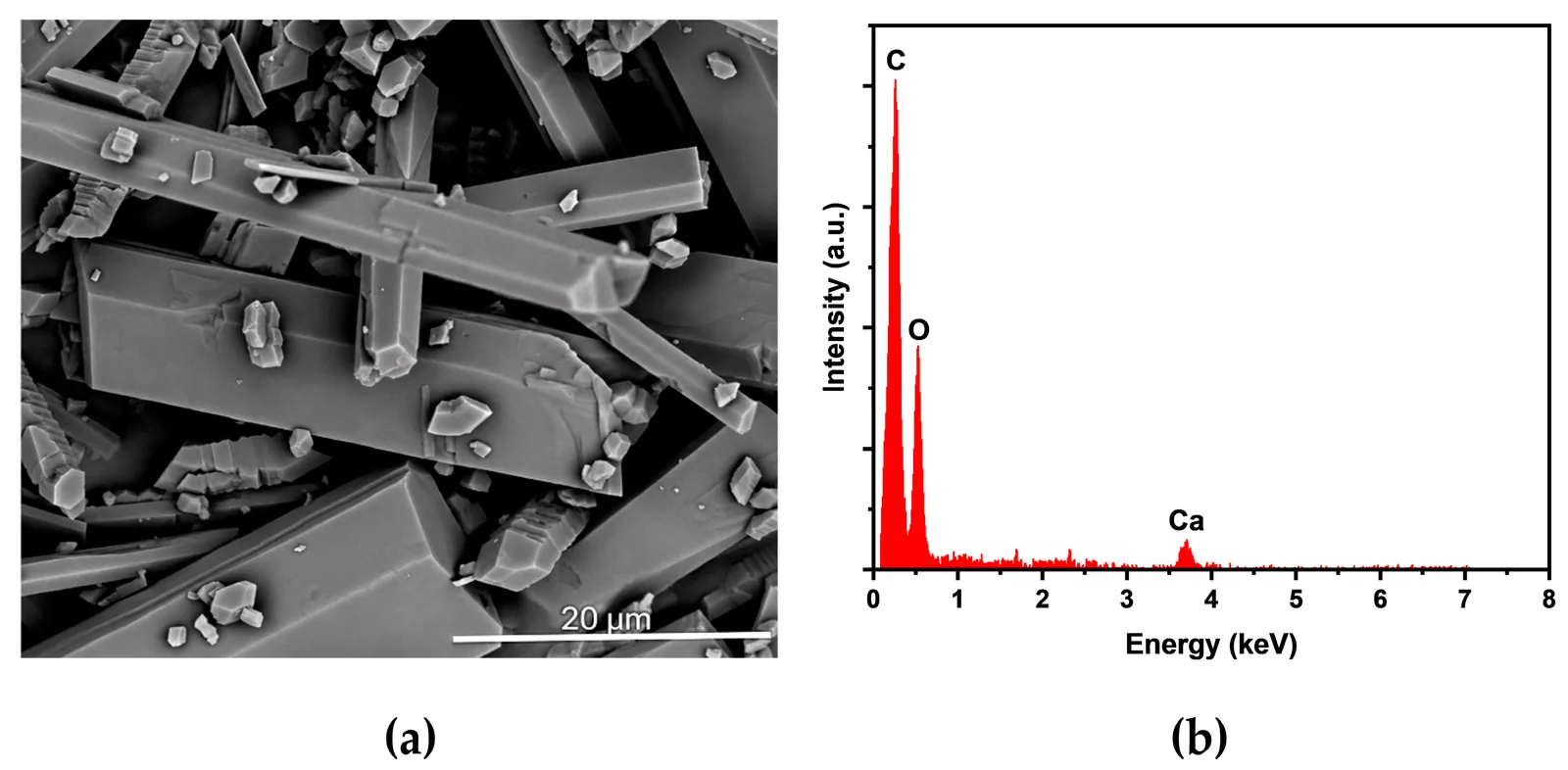
(**a**) SEM image of the Ca-BTC nanorods at 2500× magnification and (**b**) EDS spectrum.

The morphological and elemental characterization of the Ca-BTC MOF is depicted in Figure 2. The SEM image in Figure 2a, recorded at a magnification of 2500×, features well-defined Ca-BTC nanorods with a hexagonal morphology. The corresponding EDS spectrum in Figure 2b contains the characteristic peaks of the constituent elements.

Figure S1a of the Supplementary Material shows the bright-field TEM image of the Ca-BTC material, revealing irregularly shaped, closely packed nanoscale domains. These features correspond to fragments and aggregates derived from the Ca-BTC nanorods after grinding during sample preparation. The HRTEM micrograph in Figure S1b directly reveals periodic lattice fringes, providing evidence of ordered crystalline regions at the nanoscale. The coexistence of clearly resolved lattice fringes with regions of less distinct contrast suggests that crystallinity is not homogeneous throughout the entire aggregate. This is consistent with the polycrystalline character and possible local structural disorder, as further indicated by the SAED pattern in Figure S1c. The SAED pattern provides complementary evidence of the polycrystalline nature of the material, showing concentric rings with localized intensity maxima. The main rings can be correlated with the most prominent XRD reflections, particularly those at 16.60° (−111), 28.53° (0.03), and 33.27° (0.22), corresponding to interplanar spacings of approximately 5.34, 3.13, and 2.69 Å, respectively. In contrast, the two lowest-angle XRD reflections, at 10.95° and 12.15°, correspond to considerably larger interplanar spacings of 8.07 and 7.28 Å, respectively. Consequently, their corresponding electron-diffraction features are expected to occur closer to the transmitted-beam position, where their resolution may be limited by the intense central beam and the experimental detection geometry. The ring-like, rather than single-spot, character of the SAED pattern further indicates that the material consists of multiple crystalline domains with different orientations, consistent with its polycrystalline nature. In addition, the broadening and diffuse intensity of some diffraction features are consistent with local lattice distortions and microstrain rather than with a perfectly ordered crystalline lattice. Thus, XRD and SAED provide complementary structural information: XRD provides phase-level crystallographic information, while SAED demonstrates that crystalline ordering is also present within the nanoscale domains observed by HRTEM.

**Figure 3:**
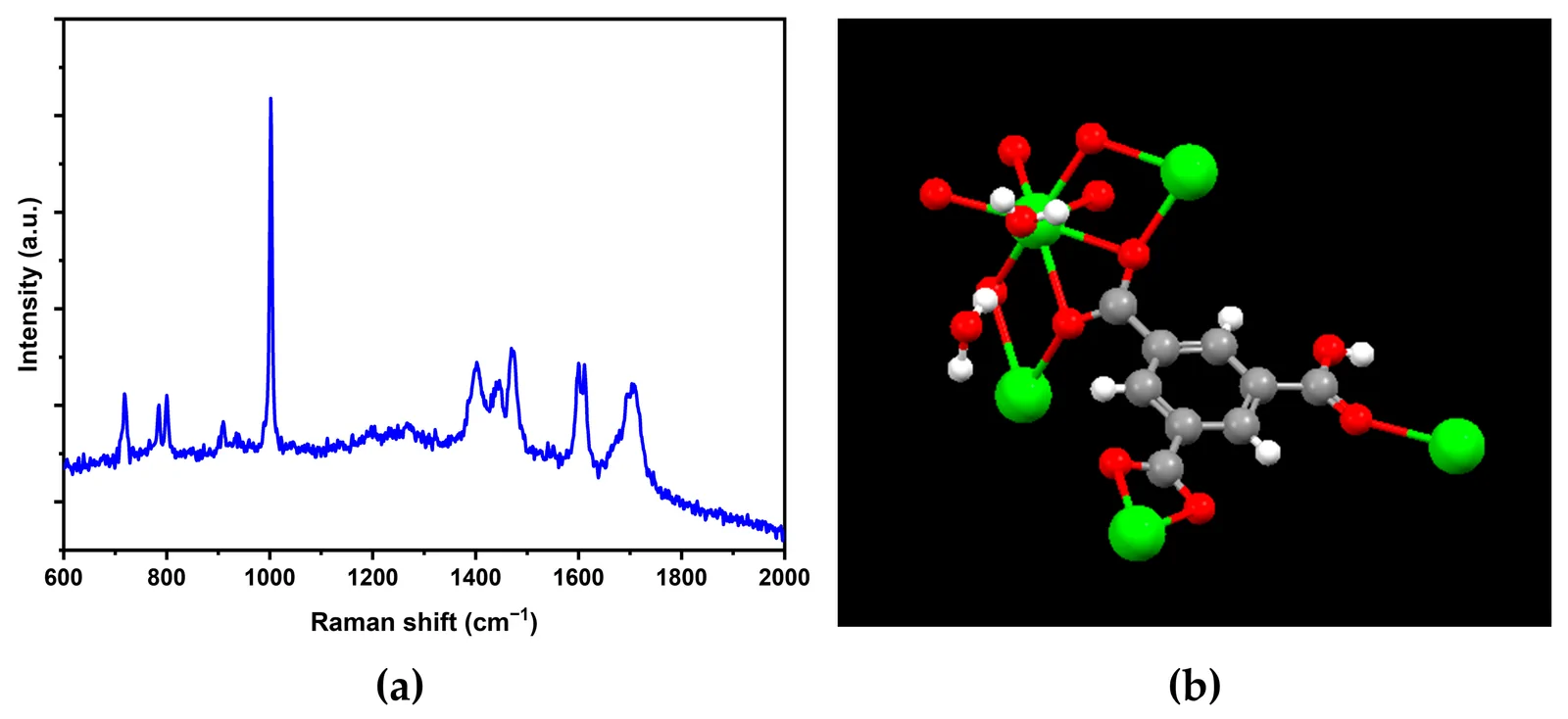
(**a**) Raman spectrum of the Ca-BTC MOF. (**b**) Molecular structure of the Ca-BTC compound: green, Ca atoms; red, O atoms; gray, C atoms; and white, H atoms.

The Raman spectrum in Figure 3a provides insight into the molecular vibrations of the Ca-BTC framework. The most intense peak, observed at 996 cm^−1^, is assigned to the Ca–O stretching mode, supporting the primary metal–oxygen coordination illustrated in Figure 3b. Other significant peaks elucidate the carboxylate linker’s bonding nature. The features at 1593 and 1405 cm^−1^ are attributed to the asymmetric and symmetric O–C–O stretching vibrations of the carboxylate groups, respectively. Their positions are consistent with the bridging coordination of the carboxylate groups to the calcium ions. Additional peaks at 745 and 786 cm^−1^ correspond to O–C–O deformation modes, while the band at 1706 cm^−1^ is assigned to C=O stretching, in agreement with prior reports [26].

### 2.2. Electrochemical Properties

**Figure 4:**
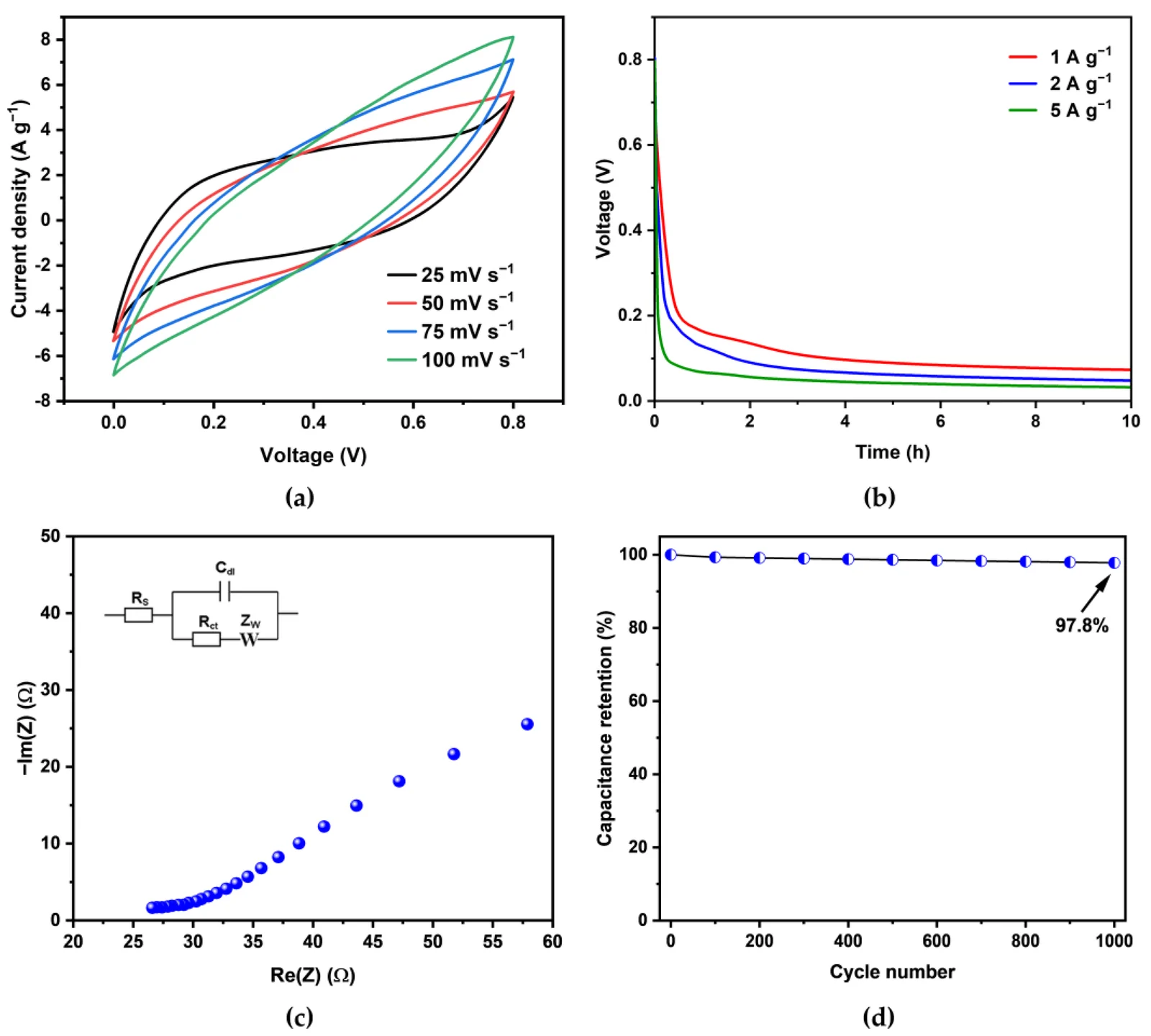
(**a**) CV curves of the Ca-BTC-based SC device at different scan rates; (**b**) GCD curves at current densities of 1, 2, and 5 A g^−1^. (**c**) Nyquist plot obtained from EIS, with the equivalent circuit model shown in the inset. (**d**) Capacitance retention during charge–discharge cycling.

Figure 4a shows the CV curves of the SC device obtained at scan rates ranging from 25 to 100 mV s^−1^. The curves exhibit a quasi-rectangular shape and become broader and slightly less rectangular as the scan rate increases, with a corresponding increase in current response. This behavior is similar to that of an electric double-layer capacitor (EDLC), without evident pseudocapacitive effects [27]. The deviation from the ideal rectangular shape at higher scan rates can be attributed to kinetic limitations and restricted ion diffusion within the electrode material.

To calculate the specific capacitance of the device from the CV data, Equation (1) was used:

(1) $$C_{\mathrm{sp}}=\frac{1}{2\cdot m\cdot ∆V\cdot v}\int I\left(V\right)\mathrm{dV},$$

where *C_sp_* is the specific capacitance in farads per gram (F g^−1^), *m* is the mass of the active material in grams (g), Δ*V* is the voltage window (V), ν is the scan rate in volts per second (V s^−1^), *I* is the current in amperes (A), and *V* is the potential in volts (V). The integral represents the integrated area of the CV curve, which is related to the charge stored in the electrode material [27]. The calculated *C_sp_* values were 296.5, 217.0, 159.0, and 112.0 F g^−1^ at scan rates of 25, 50, 75, and 100 mV s^−1^, respectively. The decrease in *C_sp_* with increasing scan rate can be attributed to the limited access of ions to the active sites at higher scan rates. For comparison, Figure S2 shows the CV curves of the reference device assembled using graphene electrodes without Ca-BTC at the same scan rates. The curves also exhibit a quasi-rectangular shape; however, they are noticeably narrower than those obtained for the Ca-BTC-based device, indicating a lower current response. The *C_sp_* values of the reference device were 157.3, 113.4, 100.6, and 91.1 F g^−1^ at scan rates of 25, 50, 75, and 100 mV s^−1^, respectively. These values are lower than those obtained for the Ca-BTC-based device at all scan rates, indicating that the incorporation of Ca-BTC enhances the charge-storage capability of the graphene-based electrode. This behavior is consistent with the reported physicochemical and electrochemical properties of Ca-MOFs, in agreement with previous studies on similar Ca-MOF systems [28,29].

Figure 4b illustrates the GCD curves of the SC device at current densities of 1, 2, and 5 A g^−1^. At the end of the 10 h discharge period, the voltage decreased progressively from 0.07 to 0.04 and 0.03 V as the current density increased from 1 to 2 and 5 A g^−1^, respectively. The initial voltage drop also became more pronounced at higher current densities due to increased ohmic losses. The similar discharge profiles at the different current densities indicate consistent electrochemical behavior of the Ca-BTC-based SC device.

The EIS results presented as Nyquist plots in Figure 4c, obtained from the EIS data and fitted using the Randles equivalent circuit shown in the inset, reveal a series resistance (*R_s_*) of 26.59 Ω and a charge-transfer resistance (*R_ct_*) of 2.63 Ω. These resistance values suggest limited ohmic losses and relatively favorable charge-transfer kinetics at the electrode/electrolyte interface [30]. Finally, the cycling stability test shown in Figure 4d demonstrates that the device retains 97.8% of its initial capacitance after 1000 charge–discharge cycles, corresponding to limited performance degradation.

### 2.3. Optical and Luminescent Properties

The UV–Vis absorption spectrum of the Ca-BTC MOF is shown in Figure S3. A broad absorption band is observed in the UV region, with a maximum at approximately 308 nm, confirming the absorption of ultraviolet radiation by the material. The luminescence properties of the Ca-BTC MOF were subsequently investigated under excitation at 265 and 365 nm.

**Figure 5:**
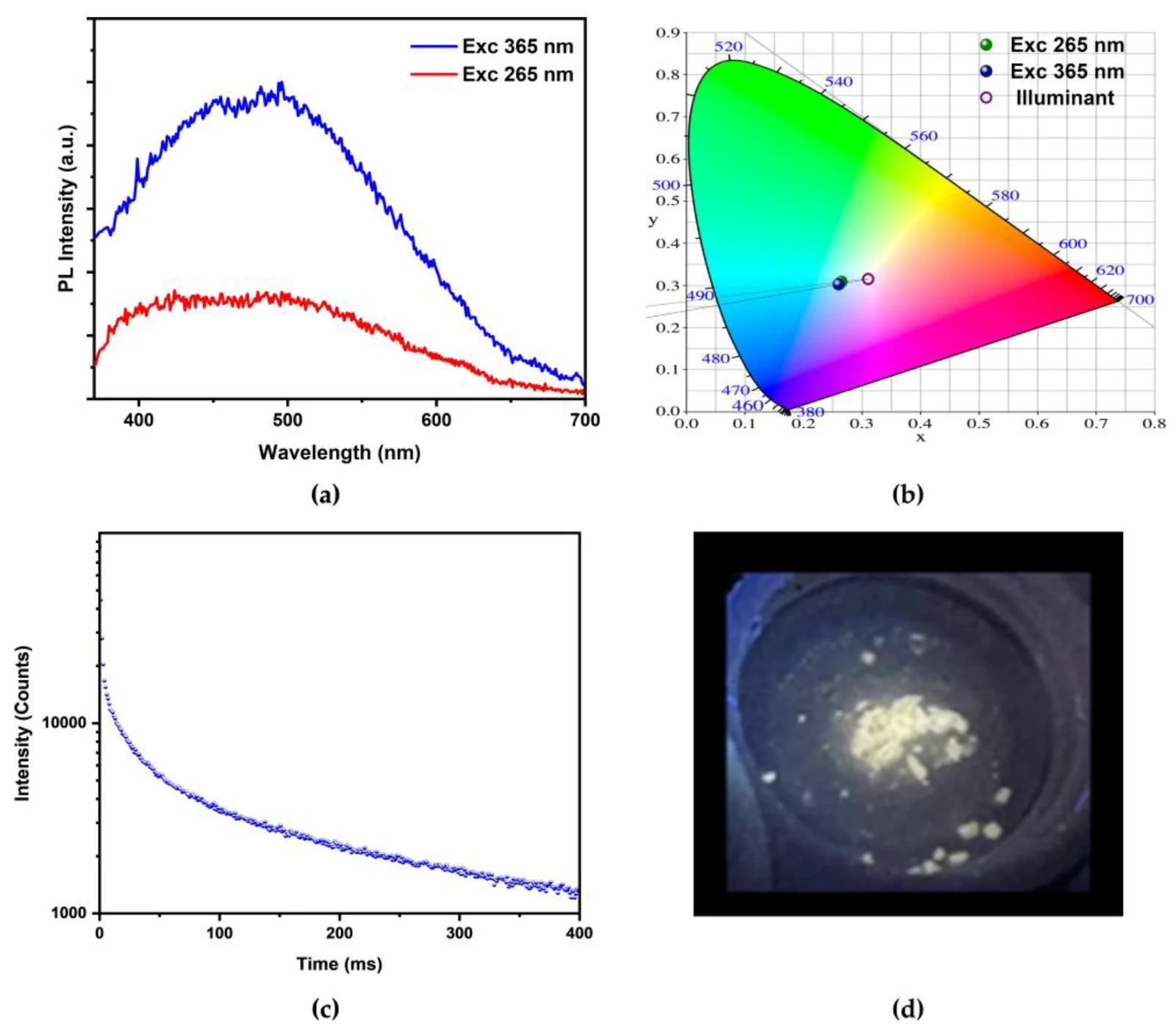
(**a**) Emission spectra under excitation at 265 and 365 nm. (**b**) CIE chromaticity diagram. (**c**) Luminescence decay curve. (**d**) Bluish-white emission under excitation with a 365 nm UV lamp.

Figure 5a exhibits the emission spectra of the Ca-BTC MOF under excitation at 265 and 365 nm. In both cases, a broad emission band extending across most of the visible region from approximately 380 to 700 nm is observed, with a maximum intensity at 490 nm, corresponding to the blue region. The emission intensity is significantly higher under 365 nm excitation, while the overall spectral profile remains similar for both excitation wavelengths. The excitation spectrum monitored at an emission wavelength of 490 nm (Figure S4) shows a broad excitation band extending from 250 to 425 nm, with a maximum around 375 nm. The broad excitation band encompasses both excitation wavelengths, which is consistent with the excitation conditions used for the emission measurements. As shown in the CIE chromaticity diagram in Figure 5b, the calculated color coordinates for 265 and 365 nm excitation are (0.2646, 0.3099) and (0.2599, 0.3038), respectively. The proximity of these coordinates indicates a consistent bluish-white emission under both excitation wavelengths. This observation is further supported by the correlated color temperatures (CCTs) of 10,347 and 10,370 K, respectively. Similar white-light emission has been reported for sodium zinc phosphate glasses doped with 0.5 mol% Tb^3+^ [24]. For comparison, the pristine BTC ligand was also evaluated under the same excitation conditions. No detectable luminescence was observed for the uncoordinated BTC ligand, indicating that the observed emission is associated with the Ca-BTC framework rather than with the pristine organic ligand.

The luminescence decay curve in Figure 5c yields an average lifetime of 51.48 ms, indicating a long-lived emission from the material. Furthermore, the material exhibits a photoluminescence quantum yield (PLQY) of 7.7%, which is comparable to the 7.4–7.8% room-temperature PLQY reported by Furman et al. [31] for the Ca-based hybrid framework Ca(FDC)(H_2_O)_2_, which contains the 9-fluorenone-2,7-dicarboxylate ligand. In that study, the enhanced luminescence was attributed to the increased structural rigidity provided by coordination of the organic ligand to the Ca-based framework, which can restrict molecular motions and reduce nonradiative relaxation pathways. Although the ligand and structural environment of the present material differ from those of Ca(FDC)(H_2_O)_2_, the comparable PLQY values suggest that framework-induced structural confinement and coordination interactions may also contribute to the observed luminescence efficiency. Finally, Figure 5d provides visual evidence of the bright bluish-white emission under excitation with a 365 nm UV lamp.

## 3. Experimental Details

Benzene-1,3,5-tricarboxylate (BTC) was used as the organic ligand for the formation of the calcium benzene-1,3,5-tricarboxylate (Ca-BTC) MOF. The synthesis methodology was adapted from the procedure reported by Medina-Velazquez et al. [32] for Eu-BTC. Calcium chloride (2.86 mmol), trimesic acid (9.29 mmol), and sodium bicarbonate were added to a reaction flask containing 30 mL of deionized water, and the mixture was stirred at 80 °C until complete dissolution of the reagents. The Ca-BTC MOF subsequently precipitated from the solution as a white solid insoluble in water. The resulting product was isolated by centrifugation, and the white precipitate was dried at 50 °C for 2 h.

X-ray diffraction (XRD) analysis was performed on a Siemens D5000 diffractometer (Karlsruhe, Germany) with CuKα radiation (λ = 1.5405 Å). The XRD patterns were recorded over the 2θ range of 10–80° at a scanning rate of 0.50° min^−1^. The morphological characterization of the MOF was carried out by scanning electron microscopy (SEM, JEOL JSM-7600F FEG, Tokyo, Japan). Elemental analysis was conducted by energy-dispersive X-ray spectroscopy (EDS) with an Oxford INCA X-Act energy-dispersive X-ray analyzer (Abingdon, UK) coupled to the SEM. Transmission electron microscopy (TEM) and high-resolution transmission electron microscopy (HRTEM) images were acquired using a JEOL JEM-ARM200F microscope (Tokyo, Japan) with a cold field-emission electron gun at a 200 kV accelerating voltage to investigate the morphology and local crystalline structure of the material, respectively. In addition, selected-area electron diffraction (SAED) patterns were obtained using the same microscope to further evaluate the crystallinity and crystallographic features of the sample. Image visualization, processing, and filtering were performed using the free edition of DigitalMicrograph software (version 3.63.5212.1, Gatan Inc.). Raman measurements were carried out with a Micro-Raman Jobin Yvon LabRAM spectrometer (Longjumeau, France) using a laser wavelength of 532 nm in the range of 600–2000 cm^−1^. The UV–Vis absorption spectrum was recorded with a Thermo Scientific Evolution 600 spectrophotometer (Madison, WI, USA) over the wavelength range of 250–700 nm. Photoluminescence measurements were conducted with a Varian Cary Eclipse Fluorescence Spectrometer (Mulgrave, Victoria, Australia) at excitation wavelengths of 265 and 365 nm. The luminescence lifetime was measured at an excitation wavelength of 365 nm while monitoring the emission at 490 nm. Excitation spectra were obtained with a Jobin–Yvon Fluorolog 3–22 spectrofluorometer (Longjumeau, France). Quantum yield measurements were performed with the same spectrofluorometer equipped with a Horiba Jobin–Yvon integrating sphere (Longjumeau, France). All measurements were conducted at room temperature.

The electrodes for the symmetric solid-state supercapacitor (SC) device were fabricated by depositing a conductive graphene-based ink onto two separate stripboards, which were used as substrates. The stripboards had dimensions of 2 × 3 cm, while the graphene layers had dimensions of 1.5 × 2.5 cm. The graphene ink was heated and pressed onto the substrates at 130 °C and 10 MPa for 25 min to improve its adhesion, resulting in a graphene loading of 13.33 mg cm^−2^ per electrode. A Ca-BTC MOF-based paste was prepared by mixing Ca-BTC MOF powder (3% *w*/*v*) and polyvinyl alcohol (PVA, 0.3% *w*/*v*) in distilled water, with PVA serving as the binder. The resulting paste was deposited onto the graphene layers using the doctor-blade method and dried at 60 °C for 12 h, resulting in a total active-material loading of 16 mg cm^−2^ per electrode, corresponding to the combined graphene and Ca-BTC loadings. Copper current collectors were subsequently attached to the graphene layers using silver paste. The SC device was then assembled in a sandwich configuration, with a filter paper separator impregnated with a phosphoric acid (H_3_PO_4_) electrolyte placed between the two electrodes, allowing ionic conduction while preventing direct electrical contact. Finally, the device was sealed with silicone polymer to mechanically secure it during electrochemical characterization. In addition, a reference SC device was fabricated using the same graphene electrodes but without the Ca-BTC MOF.

The SC device was electrochemically characterized using a Zahner-Elektrik Zennium potentiostat (Kronach, Germany). The following measurements were performed: cyclic voltammetry (CV) at scan rates ranging from 25 to 100 mV s^−1^ to evaluate the electrochemical behavior and specific capacitance of the SC; galvanostatic charge–discharge (GCD) at current densities of 1, 2, and 5 A g^−1^; and electrochemical impedance spectroscopy (EIS) over a frequency range of 0.01 Hz to 100 kHz. Additionally, the cyclic stability of the SC device was evaluated over 1000 GCD cycles, with the capacitance retention calculated every 100 cycles.

## 4. Conclusions

This study demonstrates the potential of a Ca-BTC MOF as a multifunctional material combining luminescent and electrochemical properties. The Ca-BTC MOF was successfully synthesized and characterized by XRD, SEM, EDS, HRTEM, SAED, and Raman spectroscopy, supporting the formation of the crystalline Ca_3_(BTC)_2_ phase and revealing a well-defined nanorod morphology. Photoluminescence measurements revealed a broad visible emission with a maximum in the blue region, while the CIE coordinates indicated a consistent bluish-white emission under both excitation wavelengths. Furthermore, the luminescence decay analysis yielded an average lifetime of 51.48 ms and a PLQY of 7.7%, demonstrating long-lived luminescence and highlighting the potential of the material for pc-WLED applications. Electrochemical characterization showed quasi-rectangular CV curves consistent with EDLC behavior. At 25 mV s^−1^, the Ca-BTC-based SC device achieved a *C_sp_* of 296.5 F g^−1^, which was 88.5% higher than the 157.3 F g^−1^ obtained for the reference device without Ca-BTC. EIS revealed a series resistance of *R_s_* = 26.59 Ω and a charge-transfer resistance of *R_ct_* = 2.63 Ω, indicating limited ohmic losses and relatively favorable interfacial charge-transfer characteristics. In addition, the SC device retained 97.8% of its initial capacitance after 1000 charge–discharge cycles, indicating high cycling stability and limited performance degradation. Overall, these results demonstrate the potential of Ca-BTC MOF as a multifunctional platform combining bluish-white luminescence and electrochemical charge-storage properties for optoelectronic and energy-storage applications.

## Data Availability

The datasets generated and/or analyzed during the current study are available from the corresponding author upon reasonable request.

## Supplementary Materials

The following supporting information can be downloaded at: https://www.mdpi.com/article/10.3390/molecules31214035/s1: Figure S1: (a) Bright-field TEM image, (b) HRTEM image, and (c) SAED pattern of the Ca-BTC MOF; Figure S2: CV curves of the graphene-based reference SC device without Ca-BTC at scan rates of 25, 50, 75, and 100 mV s^−1^; Figure S3: UV–Vis absorption spectrum of the Ca-BTC MOF; Figure S4: Excitation spectrum of the Ca-BTC MOF monitored at an emission wavelength of 490 nm.

## Abbreviations

The following abbreviations are used in this manuscript:

BTC	Benzene-1,3,5-tricarboxylate
Ca-BTC	Calcium Benzene-1,3,5-tricarboxylate
Ca-MOFs	Calcium-Based Metal–Organic Frameworks
CCDC	Cambridge Crystallographic Data Centre
CCT	Correlated Color Temperature
CIE	Commission Internationale de l’Éclairage (International Commission on Illumination)
CuKα	Copper K-alpha Radiation
C_sp_	Specific Capacitance
CV	Cyclic Voltammetry
EDLC	Electric Double-Layer Capacitor
EDS	Energy-Dispersive X-ray Spectroscopy
EIS	Electrochemical Impedance Spectroscopy
GCD	Galvanostatic Charge–Discharge
HRTEM	High-Resolution Transmission Electron Microscopy
MOFs	Metal–Organic Frameworks
pc-WLED	Phosphor-Converted White Light-Emitting Diode
PLQY	Photoluminescence Quantum Yield
R_ct_	Charge-Transfer Resistance
R_s_	Series Resistance
SAED	Selected-Area Electron Diffraction
SC	Supercapacitor
SEM	Scanning Electron Microscopy
TEM	Transmission Electron Microscopy
UV	Ultraviolet
XRD	X-ray Diffraction
