# Supplementary material for: Engineering Ca-BTC MOF Nanorods with Electrochemical and Luminescent Functionalities
Source: Molecules. 2026 Nov 9;31(21):4035. doi: 10.3390/molecules31214035 (PMC13765281; doi:10.3390/molecules31214035)
Supplement: Supplementary file 1 [file molecules-31-04035-s001.zip › molecules-4474472-supplementary.pdf]

Supplementary Material

Engineering Ca-BTC MOF Nanorods with Electrochemical and Luminescent Functionalities

Luis A. Garcés-Patiño [1], Esmeralda L. Martínez-Piñeiro [2], Edith Osorio-de-la-Rosa [3], Nicolás Cayetano-Castro [4], Ulises Caldiño [5], Fernando Pérez-Villaseñor [6], Mirna Valdez-Hernández [7], Marisol Hernández-Gutiérrez [8], Cristian A. Ospina-DeLaCruz [9], Ángel Castro-Agüero [6], Arturo Ortiz-Arroyo [6], Abraham Pacio-Castillo [8], and Dulce Y. Medina- Velazquez[13]*

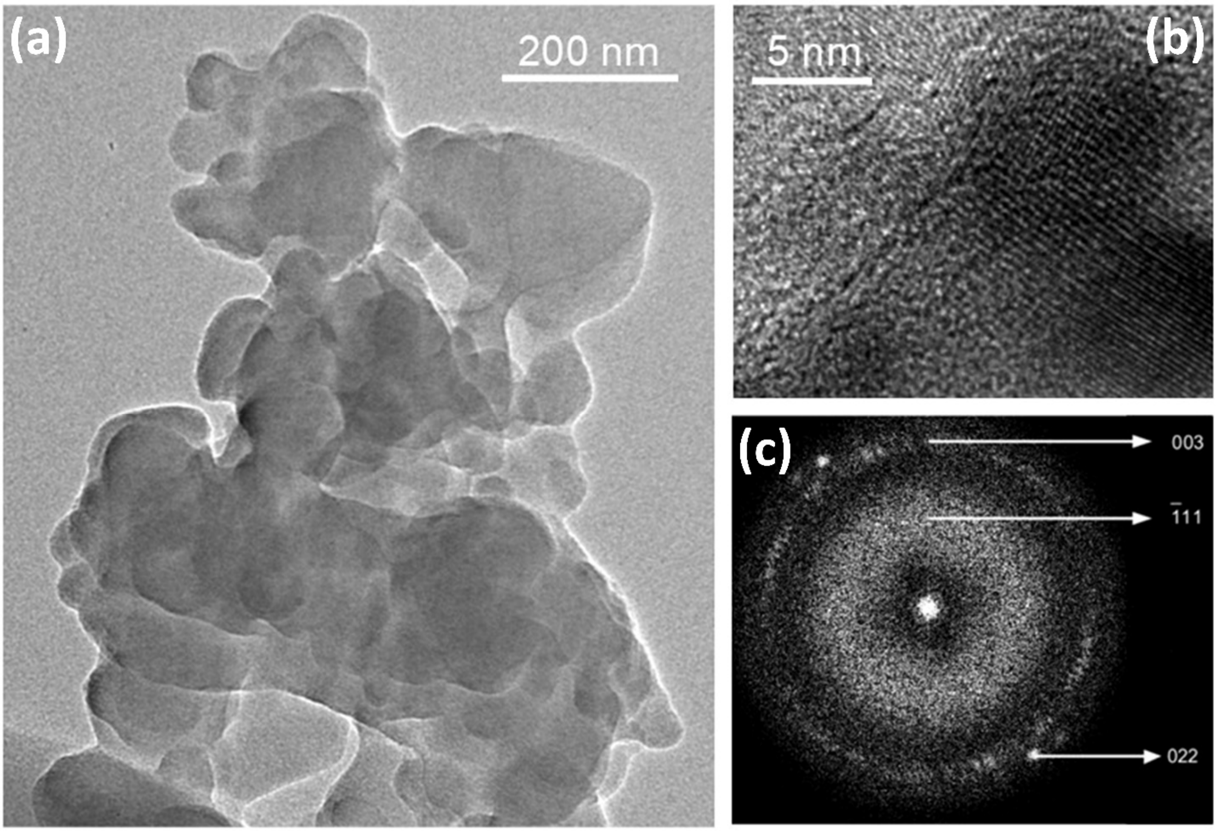

Figure S1. (**a**) Bright-field TEM image, (**b**) HRTEM image, and (**c**) SAED pattern of the Ca-BTC MOF.

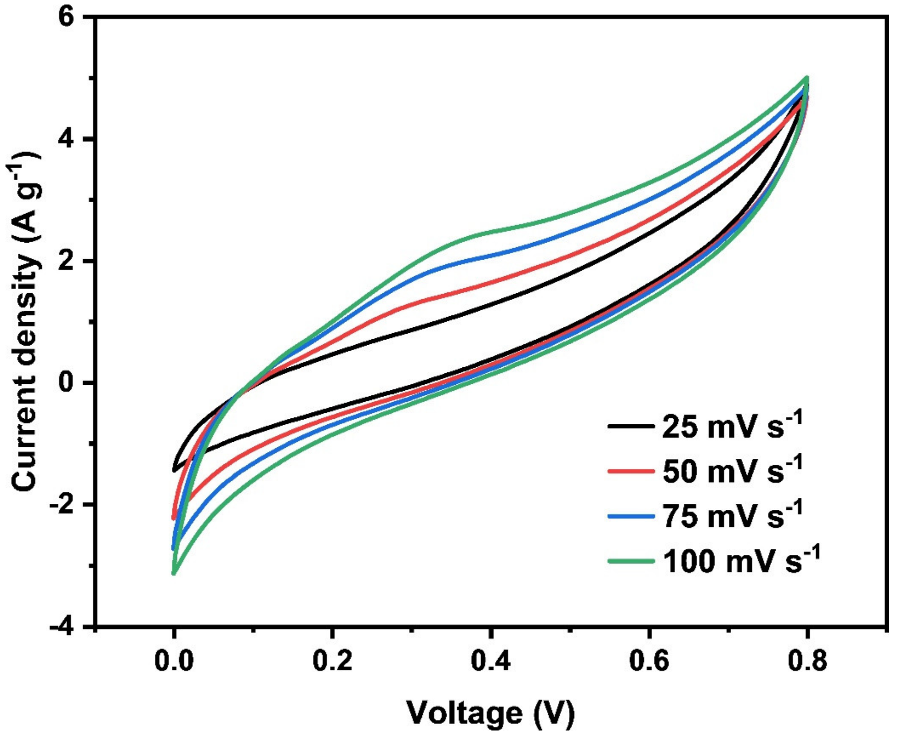

Figure S2. CV curves of the graphene-based reference SC device without Ca-BTC at scan rates of 25, 50, 75, and 100 mV s^{-1}.

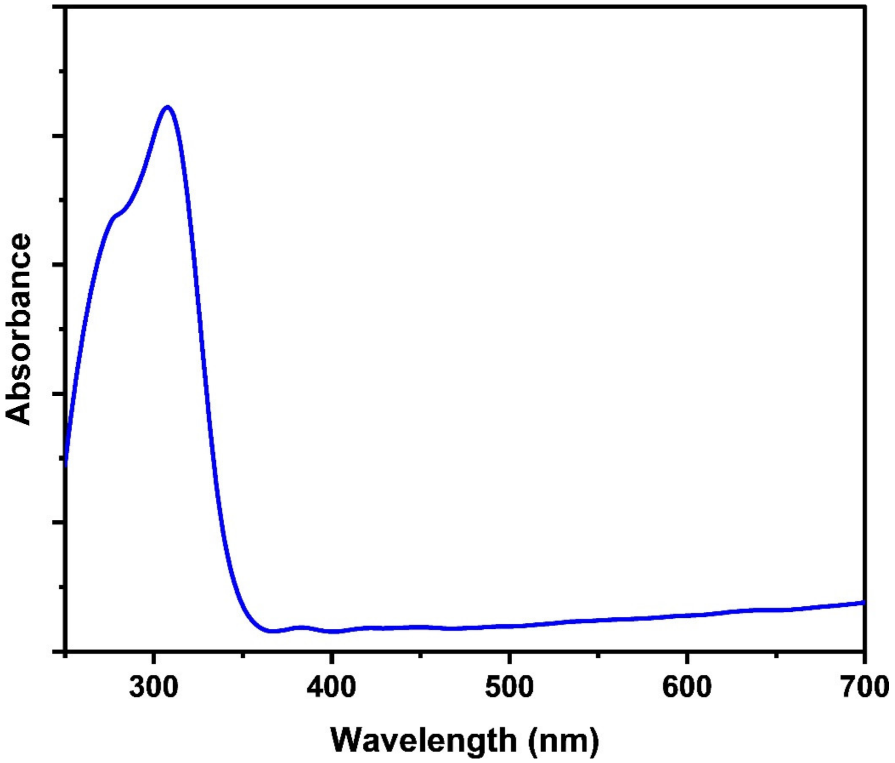

Figure S3. UV–Vis absorption spectrum of the Ca-BTC MOF.

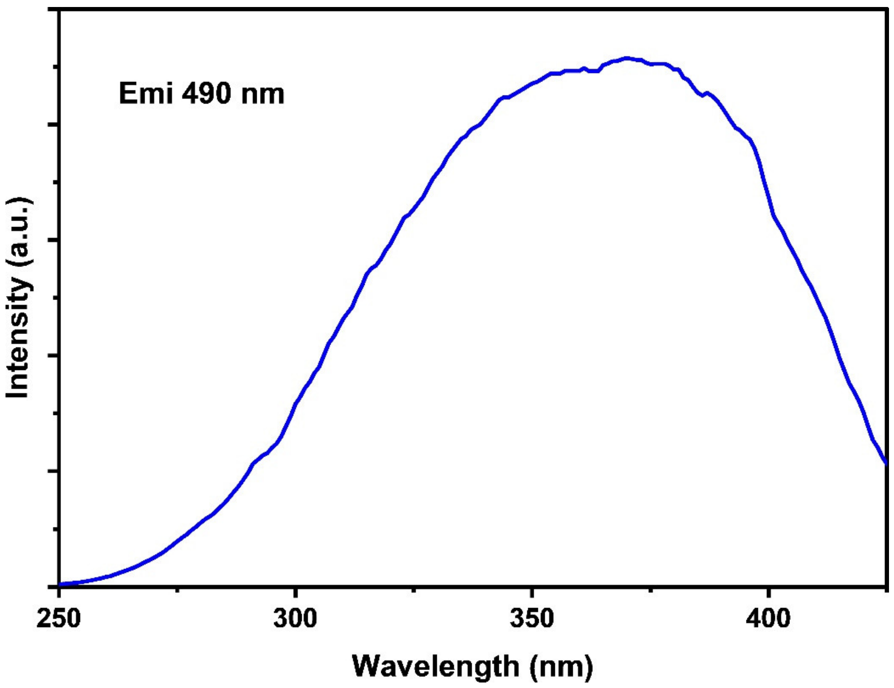

Figure S4. Excitation spectrum of the Ca-BTC MOF monitored at an emission wavelength of 490 nm.
